# The Prognostic Significance of Combined Pretreatment Fibrinogen and Neutrophil-Lymphocyte Ratio in Various Cancers: A Systematic Review and Meta-Analysis

**DOI:** 10.1155/2020/4565379

**Published:** 2020-12-09

**Authors:** Rongqiang Liu, Shiyang Zheng, Qing Yuan, Peiwen Zhu, Biao Li, Qi Lin, Wenqing Shi, Youlan Min, Qianmin Ge, Yi Shao

**Affiliations:** ^1^Department of Ophthalmology, The First Affiliated Hospital of Nanchang University, Nanchang, 330006 Jiangxi, China; ^2^Department of Hepatobiliary Surgery, The First Affiliated Hospital of Guangzhou Medical University, Guangzhou, 510220 Guangdong, China; ^3^Department of Breast Surgery, The Third Affiliated Hospital of Guangzhou Medical University, Guangzhou 510150, China

## Abstract

**Purpose:**

The prognostic value of a new scoring system, termed F-NLR, that combines pretreatment fibrinogen level with neutrophil-lymphocyte ratio has been evaluated in various cancers. However, the results are controversial. The purpose of this study was to comprehensively analyze the prognostic value of F-NLR score in patients with cancers.

**Methods:**

An integrated search of relevant studies was conducted by screening the PubMed and Embase databases. Pooled hazard ratios, with 95% confidence intervals (CIs), for overall survival (OS) and disease-free survival (DFS)/progression-free survival (PFS) were calculated to estimate the prognostic significance of F-NLR score in patients with various tumors. A random effects model was used for comprehensive analysis, and subgroup and meta-regression analyses were used to explore sources of heterogeneity.

**Results:**

Thirteen articles reporting data from of 4747 patients were included in the study. Pooled analysis revealed that high F-NLR score was significantly associated with poor OS (HR = 1.77; 95% CI, 1.51–2.08) and poor DFS/PFS (HR = 1.63; 95% CI, 1.30–2.05). Subgroup and meta-regression analyses did not alter the prognostic role of F-NLR score in OS and DFS/PFS.

**Conclusions:**

Increased F-NLR score is significantly associated with poor prognosis in patients with cancers and can serve as an effective prognostic indicator.

## 1. Introduction

Increasing evidence suggests that tumor progression is closely associated with inflammatory responses [1–4]. The systemic proinflammatory effects of tumors are considered to be the result and cause by inhibiting apoptosis and promoting angiogenesis and DNA damage [3]. Numerous inflammatory indices, including the systemic immune inflammation index, platelet/lymphocyte ratio, prognostic nutritional index, modified Glasgow prognostic score, and C-reactive protein/albumin ratio, are closely related to prognosis in patients with cancer [5–10]. Fibrinogen is a 340 kDa glycoprotein synthesized by hepatocytes and has an important role in the coagulation process and can be converted to fibrin by activated thrombin. Fibrinogen can promote tumor cell proliferation, angiogenesis, and hematogenous metastasis, and elevated serum fibrinogen levels are associated with poor prognosis in patients with various tumors [11–16]. The neutrophil-to-lymphocyte ratio (NLR) is another useful marker for assessment of the inflammatory response, which is calculated by dividing the neutrophil count by the lymphocyte count. A number of studies have reported that elevated NLR is associated with poor prognosis in patients with various malignancies, including hepatocellular carcinoma, biliary tract cancer, esophageal cancer, prostate cancer, and colon cancer [17–21]. In recent years, a new scoring system, termed F-NLR, that combines pretreatment fibrinogen levels with NLR has gradually become a hot topic. In general, F-NLR score is classified into three groups based on the cutoff value of plasma fibrinogen and NLR, and prognostic situation of patients with cancer is assessed according to different groups. At present, the F-NLR score has been reported as a promising prognostic marker in patients with different types of cancers [22–34]. However, the majority of these studies had small sample sizes; hence, the results are somewhat unreliable and controversial.

Here, we conducted a meta-analysis to comprehensively assess the role of F-NLR score in predicting prognosis in patients with cancer. We also discuss whether F-NLR score is a suitable prognostic marker for patients with cancers.

## 2. Materials and Methods

### 2.1. Search Strategy

We searched for articles in the PubMed and Embase databases until June 2019, using the keywords “fibrinogen and neutrophil-lymphocyte ratio.” Titles, abstracts, full texts, and reference lists were carefully screened to identify objective studies. We searched using keywords, without any restrictions, and manually screened the reference lists in identified publications.

### 2.2. Study Selection

Articles were considered eligible if they met the following criteria: (1) they investigated associations of F-NLR score with survival outcome in patients with any type of cancer; (2) they provided sufficient data to allow calculation of hazard ratio (HR) and 95% CI. The exclusion criteria were as follows: (1) they provided insufficient data to allow calculation of HR and 95% CI; (2) they were case reports, animal studies, reviews, letters, abstracts, or non-English language publications.

### 2.3. Data Extraction and Quality Assessment

Two authors extracted the data independently. The relevant information included the first author's name, publication year, country, tumor type, tumor stage, sample size, mean age, analysis type, sex, cutoff value, overall survival (OS), disease-free survival (DFS)/progression-free survival (PFS), hazard ratio, and the corresponding 95% CI. If a study reported the results of both univariate and multivariate analyses, those from multivariate analysis were selected, as this approach considers confounding factors and is more accurate. Each study was assessed for quality, according to the Newcastle-Ottawa Quality Assessment Scale (NOS) [35]. This study does not require the approval of the ethics committee.

### 2.4. Statistical Analysis

HR and corresponding 95% CI were used to analyze pooled data. If these statistical variables were described in the study, we used them directly in our analysis; otherwise, data were extracted from graphical survival plots. Data extracted from Kaplan-Meier survival curves were read using Engauge Digitizer version 4.1. A random effects model was used if *I*^2^ > 50%; otherwise, a fixed-effects model was used. Subgroup analysis and metaregression were used to explore factors contributing to heterogeneity. Publication bias was analyzed by funnel plot. All analyses were performed using STATA version 12.0 software (Stata Corporation, College Station, TX, USA). *P* values < 0.05 were considered statistically significant, unless otherwise specified.

## 3. Results

### 3.1. Study Characteristics

**Figure 1:**
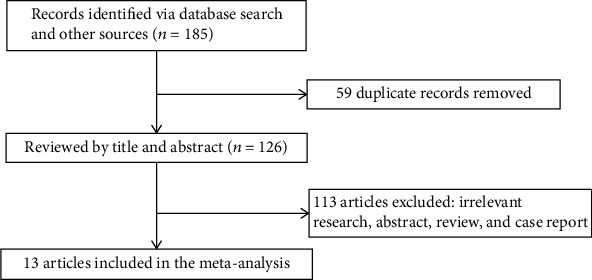
Flow diagram for study screening and selection processes.

Initial database searching resulted in the retrieval of 185 articles. Titles and abstracts were screened, and abstracts, conference articles, animal experiment studies, and studies reporting incomplete data were excluded. Eventually, 13 articles analyzing the relationship between F-NLR score and outcomes of patients with various cancers were identified. All included articles were published until June 2019. The flow chart for study identification is presented in Figure 1. The total number of patients in the included articles was 4747, with numbers per study ranging from 68 to 1293. In all studies, fibrinogen levels and neutrophil and lymphocyte counts were detected in blood samples. Twelve studies presented OS data and six studies DFS/PFS data. Seven studies were conducted in China, five in Japan, and one in Italy. All studies reported a significant association between high F-NLR score and poor prognosis. The quality of included articles ranged from 6 to 7 (NOS; mean = 6.6). Basic information from all included studies is presented in Table 1.

### 3.2. F-NLR Score and OS

**Figure 2:**
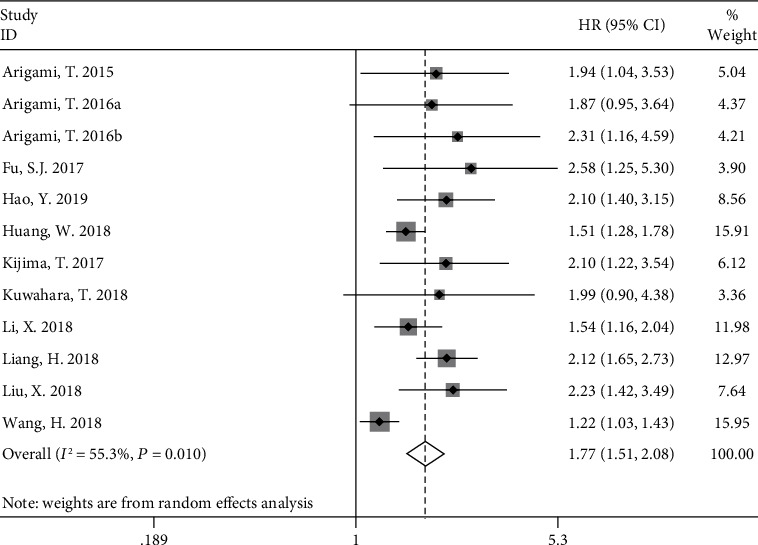
Forest plot of the relationship between F-score and OS.

Twelve studies used OS to describe the relationship between F-score and prognosis. The results of the meta-analysis exhibited moderate heterogeneity (*P* = 0.010, *I*^2^ = 55.3%); therefore, we used a random effects model to calculate the pooled HR (95% CI). The results of meta-analysis revealed that high F-NLR score was significantly associated with poor OS, with a pooled HR of 1.77 (95% CI, 1.51–2.08) (Figure 2).

### 3.3. F-NLR Score and DFS/PFS

**Figure 3:**
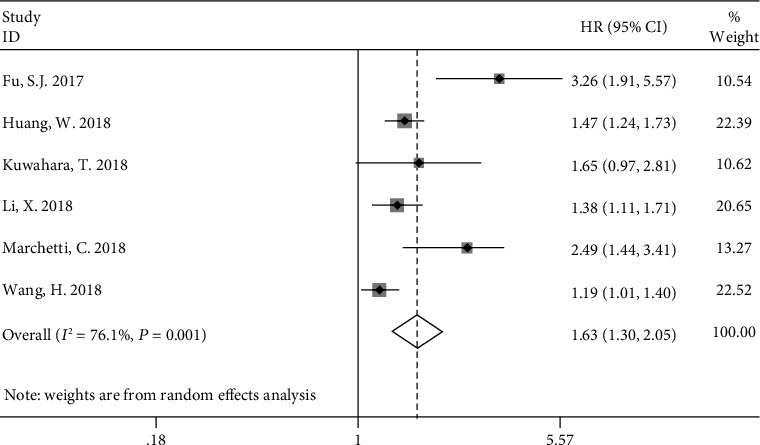
Forest plot of the relationship between F-score and DFS/PFS.

Six studies also reported DFS/PFS data to document the relationship between F-score and prognosis. We used a random effects model to calculate pooled HR (95% CI) values, based on the detection of clear heterogeneity among the six studies (*P* = 0.001, *I*^2^ = 76.1%). The results showed that high F-NLR score was significantly associated with poor DFS/PFS, with a pooled HR of 1.63 (95% CI, 1.30–2.05) (Figure 3).

### 3.4. Subgroup and Metaregression Analyses

Given the existence of significant heterogeneity among the studies, we performed subgroup and regression analyses according to tumor type, publication year, sample size, country, analysis type, sex, and mean age. Subgroup and metaregression analyses did not alter the prognostic role of F-NLR score in OS and DFS/PFS. We noted slighter heterogeneity in stratified studies according to digestive system tumor, publication year (<2017), sample size (≤390), country (Japan), univariate analysis, or sex (female ≤ 31%) in OS (Table 2). Similarly, we observed no heterogeneity in stratified studies according to age (≤60) and minor heterogeneity in stratified studies with sample size in DFS/PFS (Table 3).

### 3.5. Sensitivity Analysis and Publication Bias

**Figure 4:**
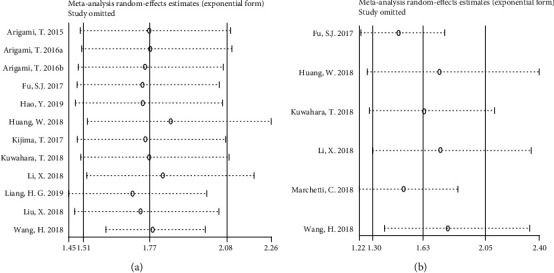
Funnel plot of sensitivity analysis. (a) Sensitivity analysis for OS. (b) Sensitivity analysis for DFS/PFS.

**Figure 5:**
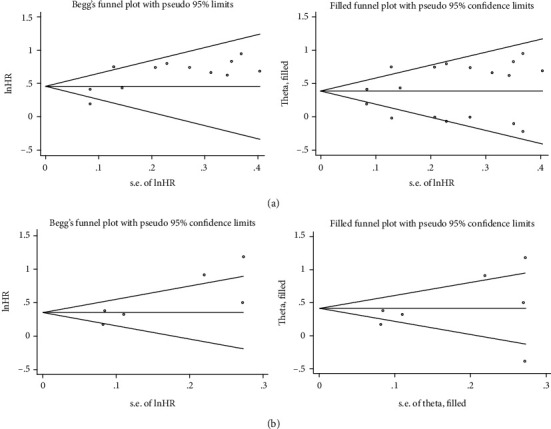
Funnel plots for publication bias. (a) Funnel plots without and with trim and fill to evaluate OS data. (b) Funnel plots without and with trim and fill to evaluate DFS/PFS data.

Sensitivity analysis was conducted by removing each study from the combined dataset sequentially. As shown in Figure 4, the results did not differ significantly from the overall analysis, indicating that the outcome was stable. Further, we used funnel plots to evaluate publication bias using total OS or DFS/PFS data. Begg's test and Egger's test were applied to provide statistical evidence for funnel plot symmetry. As shown in Figure 5, Begg's test and Egger's test *P* values were 0.537 and 0.01 for OS and 0.06 and 0.047 for DFS/PFS, indicating there existed publication bias for either type of report. However, the trim-and-fill method displayed that the pooled HR for OS was 1.47 (95% CI: 1.263-1.732) and 1.58 (95% CI: 1.187-1.916) for DFS/PFS, which further confirmed the results were not affected.

## 4. Discussion

The F-NLR score, based on fibrinogen levels and neutrophil and lymphocyte counts, represents inflammatory responses and the cancer microenvironment. Fibrinogen, an acute-phase response protein, is primarily synthesized in the liver but can also be produced by tumor cells [36]. In particular, tumor cells can promote fibrinogen secretion by producing interleukin-6 [37]. When stimulated with inflammatory factors or by tumors, activated thrombin can transform fibrinogen into fibrin, which can form a stable framework and extracellular matrix around tumor cells, preventing tumor cell killing by immune cells [38]. Further, in vivo tumor metastasis is remarkably diminished in fibrinogen-deficient mice [39]. Neutrophils can promote tumor invasion, metastasis, and angiogenesis by producing various cytokines, such as tumor necrosis factor-alpha, vascular endothelial growth factor, fibroblast growth factor, angiopoietin, and interleukin [40, 41]. Neutrophils can also promote tumor cell metastasis by secreting neutrophil extracellular traps [42]. In addition, elevated neutrophil numbers can prevent other immune cells from killing tumor cells, and high levels of tumor-infiltrating neutrophils are an indicator of poor prognosis in gastric cancer [43]. Lymphocytes have important roles in antitumor immune defense, and lymphocyte reduction can be considered an immune deficiency, in terms of antitumor immune responses. Lymphocytes can promote tumor cell apoptosis and produce cytokines to inhibit tumor cell proliferation and metastasis [44, 45]. A previous study reported that lymphopenia is associated with poor prognosis [46]. Studies have confirmed that fibrinogen or NLR can present a good prognostic indicator for patients with cancer. However, fibrinogen or NLR alone may emerge a limited effect on tumor progression. F-NLR score overcomes the unfavorable effect of fibrinogen and NLR and effectively improves the predicted value for patients with cancers.

Liu et al. analyzed 1293 consecutive patients who underwent curative surgery for gastric cancer. They found that higher F-NLR scores were associated with larger tumor size, deeper tumor invasion, and more lymph node metastasis and were independently prognostic predictor [32]. Similarly, Arigami et al. revealed that the F-NLR score correlated with the depth of tumor invasion, lymph node metastasis, lymphatic vessel invasion, tumor size, and stage and was an independent prognostic factor for esophageal squamous cell carcinoma [22]. Wang et al. disclosed that the 5-year DFS rates in F-NLR groups 0, 1, and 2 were 46.7%, 36.4%, and 30.1% and the 5-year overall survival (OS) rates in the above three groups were 52.0%, 39.8%, and 32.1%, respectively. They thought that lung cancer patients with a high-risk preoperative F-NLR group scores may benefit from adjuvant therapy by subgroup analysis [34]. Li et al. showed that an elevated F-NLR score was significantly associated with worse OS and DFS in patients with colorectal adenocarcinoma. They also found that DFS in a low F-NLR score group was significantly shortened after chemotherapy, and patients with a relatively high F-NLR score group showed a slight OS benefit from adjuvant chemotherapy [30].

To the best of our knowledge, this study is the first meta-analysis to comprehensively explore the prognostic value of F-NLR score in various tumors. Our meta-analysis demonstrates that high F-NLR score is significantly associated with poor OS, with a pooled HR of 1.77 (95% CI, 1.51–2.08) and poor DFS/PFS (HR = 1.63; 95% CI, 1.30–2.05). Subgroup and metaregression analyses revealed that tumor type, publication year, sample size, country, analysis type, and sex may be sources of heterogeneity in OS, and sample size and age contributed to the heterogeneity in DFS/PFS. Sensitivity analysis and the trim-and-fill method showed that the pooled results were stable.

Our meta-analysis has limitations. First, all included articles were from studies with small sample sizes; hence, their results may be somewhat unreliable. Second, the research methods and cutoff values used in included studies differed from one another, which influences judgment of F-NLR score as a prognostic marker. Third, the majority of studies included in the meta-analysis were conducted in three countries, which may affect the reliability of our results. Fourth, we acknowledged that there existed the flaw that only two database engines were used to retrieve data. Fifth, extracting HR and 95% CI from the survival curve may bring slight error. Finally, due to the lack of the additional clinicopathological parameters of selected articles, we cannot assess the relationship between F-NLR scores and other clinicopathological parameters.

In conclusion, our meta-analysis demonstrates that increased F-NLR score is significantly associated with poor prognosis in patients with cancer and that F-NLR score can be used as an effective prognostic indicator. However, considering the limitations of this study, further large-scale, well-designed, and multicenter prospective researches are needed to validate our results before the application of F-NLR score for the prognosis of various cancers.

## Figures and Tables

**Table 1:** The basic information of included studies.

Study (year)	Tumor type	Region	No. of patients	Age (mean year)	Cancer stage or grade	Definition of F-NLR score	End point	Quality score
Arigami et al. (2015)	ESCC	Japan	238	65	Stage I-III	2 = F > 400 mg/dL & NLR > 3.0 1 = F > 400 mg/dL or NLR > 3.0 0 = F ≤ 400 mg/dL and NLR ≤ 3.0	OS^∗^	7
Arigami et al. (2016a)	GC	Japan	275	66	Stage I-IV	2 = F > 400 mg/dL & NLR > 3.0 1 = F > 400 mg/dL or NLR > 3.0 0 = F ≤ 400 mg/dL and NLR ≤ 3.0	OS^∗^	7
Arigami et al. (2016b)	GC	Japan	68	66	NR	2 = F > 400 mg/dL & NLR > 3.0 1 = F > 400 mg/dL or NLR > 3.0 0 = F ≤ 400 mg/dL and NLR ≤ 3.0	OS	6
Fu et al. (2017)	HCC	China	130	49.5	NR	2 = F > 234.5 mg/dL & NLR > 1.84 1 = F > 234.5 mg/dL or NLR > 1.84 0 = F ≤ 234.5 mg/dL and NLR ≤ 1.84	OS, DFS	6
Hao et al. (2019)	Glioblastoma	China	187	55	NR	2 = F > 340 mg/dL & NLR > 4.1 1 = F > 340 mg/dL or NLR > 4.1 0 = F ≤ 340 mg/dL and NLR ≤ 4.1	OS^∗^	7
Huang et al. (2018)	NSCLC	China	589	60	Stage I-IIIA	2 = F > 348 mg/dL & NLR > 2.3 1 = F > 348 mg/dL or NLR > 2.3 0 = F ≤ 348 mg/dL and NLR ≤ 2.3	OS^∗^, DFS^∗^	7
Kijima et al. (2017)	ESCC	Japan	98	64.9	Stage III-IV	2 = F > 400 mg/dL & NLR > 3.0 1 = F > 400 mg/dL or NLR > 3.0 0 = F ≤ 400 mg/dL and NLR ≤ 3.0	OS^∗^	7
Kuwahara et al. (2018)	HPC	Japan	111	67	Stage III-IV	2 = F > 341 mg/dL & NLR > 3.59 1 = F > 341 mg/dL or NLR > 3.59 0 = F ≤ 341 mg/dL and NLR ≤ 3.59	OS, PFS	7
Li et al. (2018)	CRC	China	693	NR	Stage I-III	2 = F > 297 mg/dL & NLR > 2.34 1 = F > 297 mg/dL or NLR > 2.34 0 = F ≤ 297 mg/dL and NLR ≤ 2.34	OS, DFS	6
Liang et al. (2019)	NSCLC	China	456	61	Stage I-IIIA	2 = F > 377 mg/dL & NLR > 2.28 1 = F > 377 mg/dL or NLR > 2.28 0 = F ≤ 377 mg/dL and NLR ≤ 2.28	OS	6
Liu et al. (2018)	GC	China	1293	59	Stage I-III	2 = F > 400 mg/dL & NLR > 5.0 1 = F > 400 mg/dL or NLR > 5.0 0 = F ≤ 400 mg/dL and NLR ≤ 5.0	OS^∗^	7
Marchetti et al. (2018)	OC	Italy	94	55	Stage I-IV	2 = F > 450 mg/dL & NLR > 3.24 1 = F > 450 mg/dL or NLR > 3.24 0 = F ≤ 450 mg/dL and NLR ≤ 3.24	PFS	6
Wang et al. (2018)	NSCLC	China	515	60.4	Stage I-IIIA	2 = F > 338 mg/dL & NLR > 2.21 1 = F > 338 mg/dL or NLR > 2.21 0 = F ≤ 338 mg/dL and NLR ≤ 2.21	OS^∗^, DFS^∗^	7

ESCC: esophageal squamous cell carcinoma; GC: gastric cancer; HCC: hepatocellular carcinoma; NSCLC: non-small-cell lung cancer; HPC: hypopharyngeal carcinoma; CRC: colorectal adenocarcinoma; OC: ovarian cancer; OS: overall survival; DFS: disease-free survival; PFS: progression-free survival; ^∗^multivariate analysis.

**Table 2:** Subgroup analysis and metaregression of the studies reporting the effect of F-NLR score in OS.

Stratified study	No. of studies	Pooled HR (95% CI)	Heterogeneity		Metaregression		
			*I* ^2^ (%)	*P* value	Tau^2^	Adj *R*^2^ (%)	*P* value
*Cancer type*					0.04	-11.81	0.77
Digestive system	7	1.817 (1.497-2.206)	0	0.77			
NSCLC	4	1.65 (1.266-2.15)	81.9	<0.01			
Other type	1	2.103 (1.401-3.156)	—	—			
*Publication year*					0.03	15.36	0.23
≥2017	7	1.683 (1.389-2.04)	69.3	<0.01			
<2017	5	2.121 (1.598-2.817)	0	0.97			
*Sample size*					0.03	29.73	0.11
*n* ≤ 390	7	2.105 (1.684-2.631)	0	0.99			
*n* > 390	5	1.618 (1.307-2.004)	76	<0.01			
*Country*					0.03	8.8	0.36
China	7	1.715 (1.406-2.092)	71.6	<0.01			
Japan	5	2.04 (1.531-2.718)	0	0.99			
*Analysis type*					0.03	6.9	0.37
Multivariate	7	1.676 (1.371-2.049)	58.9	0.02			
Univariate	5	1.909 (1.608-2.267)	0	0.43			
*Gender (Female, %)*					0.02	44.22	0.1
>31	7	1.617 (1.35-1.937)	56.7	0.03			
≤31	5	2.12 (1.739-2.585)	0	0.98			
*Age (year)*					0.04	-12.82	0.61
>60	8	1.742 (1.389-2.184)	61.4	0.01			
≤60	4	1.874 (1.445-2.432)	46.7	0.13			

**Table 3:** Subgroup analysis and metaregression of the studies reporting the prognostic role of F-NLR score in DFS/PFS.

Stratified study	No. of studies	Pooled HR (95% CI)	Heterogeneity		Metaregression		
			*I* ^2^ (%)	*P* value	Tau^2^	Adj *R*^2^ (%)	*P* value
*Cancer type*					0.13	-35.24	0.9
Digestive system	3	1.875 (1.133-3.102)	76.6	0.01			
NSCLC	2	1.32 (1.076-1.619)	68.1	0.08			
Other type	1	2.49 (1.618-3.832)	—	—			
*Publication year*					0.04	63.11	0.1
≥2017	5	1.471 (1.22-1.773)	64.9	0.02			
<2017	1	3.26 (1.909-5.567)	—	—			
*Sample size*					0.01	93.11	0.03
*n* ≤ 390	3	2.383 (1.659-3.422)	37.3	0.2			
*n* > 390	3	1.334 (1.169-1.523)	39	0.19			
*Country*					0.07	25.86	0.34
China	4	1.497 (1.182-1.896)	71.6	<0.01			
Japan	1	1.65 (0.969-2.808)	—	—			
Italy	1	2.49 (1.618-3.832)	—	—			
*Analysis type*					0.07	21.85	0.21
Multivariate	2	1.32 (1.076-1.619)	68.1	0.08			
Univariate	4	2.005 (1.327-3.031)	75.6	<0.01			
*Gender (female, %)*					0.06	33.39	0.25
>31	4	1.46 (1.189-1.793)	72.6	0.01			
≤31	2	2.318 (1.189-4.517)	68	0.08			
*Age (year)*					0.06	30.42	0.16
>60	3	1.278 (1.12-1.459)	82.9	<0.01			
≤60	3	2.181 (1.304-3.646)	5.8	0.34			

## Data Availability

All data are in the manuscript and can be obtained from the corresponding author.
